# *QuickStats:* Percentage* of Adults Aged ≥25 Years Who Had Seen a Health Care Professional in the Past 12 Months and Who Easily Understood Information from Their Health Care Providers Most or All of the Time,^†^ by Sex and Education Level — National Health Interview Survey,^§^ United States, 2017

**DOI:** 10.15585/mmwr.mm6845a6

**Published:** 2019-11-15

**Figure:**
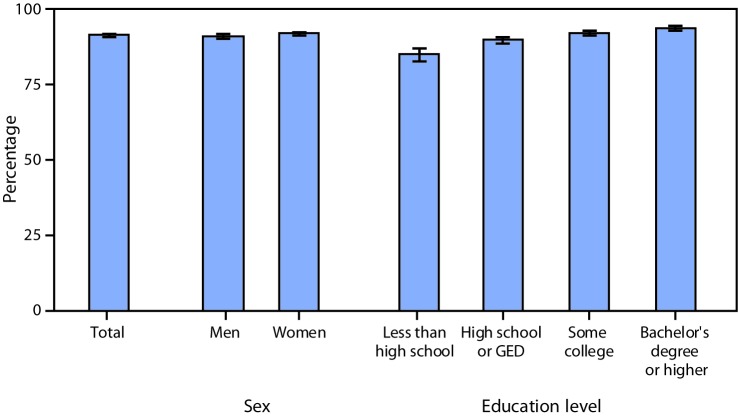
In 2017, 91.6% of adults aged ≥25 years easily understood information from their health care providers most or all of the time. The percentage of adults who easily understood health care information most or all of the time increased as education level increased. Adults who had completed a bachelor’s degree or higher were the most likely to understand their health care providers at least most of the time (93.9%), whereas those without a high school diploma were the least likely (85.2%). Men (91.0%) were somewhat less likely than women (92.1%) to have easily understood information from providers most or all of the time.

**Source:** National Health Interview Survey, 2017. https://www.cdc.gov/nchs/nhis.htm.

